# Effects of Turmeric Extract on Age-Related Skeletal Muscle Atrophy in Senescence-Accelerated Mice

**DOI:** 10.3390/life13040941

**Published:** 2023-04-03

**Authors:** Weida Lyu, Marika Kousaka, Huijuan Jia, Hisanori Kato

**Affiliations:** 1 Health Nutrition, Graduate School of Agricultural and Life Sciences, The University of Tokyo, Hongo 7-3-1, Bunkyo-ku, Tokyo 113-0032, Japan; 2 Department of Geriatric Medicine, Graduate School of Medicine, The University of Tokyo, Hongo 7-3-1, Bunkyo-ku, Tokyo 113-0032, Japan

**Keywords:** turmeric extract, age-related skeletal muscle atrophy, sarcopenia, senescence-accelerated mouse model, GR-FoxO signaling pathway

## Abstract

Muscle atrophy is one of the main causes of sarcopenia—the age-related loss of skeletal muscle. In this study, we investigated the effect of turmeric (*Curcuma longa*) extract (TE) supplementation on age-related muscle atrophy in a senescence-accelerated mouse model and explored the underlying mechanisms. Twenty-six-week-old male, senescence-accelerated mouse resistant (SAMR) mice received the AIN-93G basal diet, while twenty-six-week-old male, senescence-accelerated mouse prone 8 (SAMP8) mice received the AIN-93G basal diet or a 2% TE powder-supplemented diet for ten weeks. Our findings revealed that TE supplementation showed certain effects on ameliorating the decrease in body weight, tibialis anterior weight, and mesenteric fat tissue weight in SAMP8 mice. TE improved gene expression in the glucocorticoid receptor-FoxO signaling pathway in skeletal muscle, including *redd1*, *klf15*, *foxo1*, *murf1*, and *mafbx*. Furthermore, TE might have the certain potential on improving the dynamic balance between anabolic and catabolic processes by inhibiting the binding of glucocorticoid receptor or FoxO1 to the glucocorticoid response element or FoxO-binding element in the MuRF1 promoter in skeletal muscle, thereby promoting muscle mass and strength, and preventing muscle atrophy and sarcopenia prevention. Moreover, TE may have reduced mitochondrial damage and maintained cell growth and division by downregulating the mRNA expression of the genes *mfn2* and *tsc2*. Thus, the results indicated TE’s potential for preventing age-related muscle atrophy and sarcopenia.

## 1. Introduction

Sarcopenia is characterized as an age-related decrease of skeletal muscle mass and strength with impaired physical functioning and is mostly caused by skeletal muscle atrophy [1]. Muscle atrophy causes physical dysfunction and makes it challenging for people to carry out even normal daily activities. The mechanism behind muscle atrophy involves the degradation of contractile proteins and cellular organelles within the muscle tissue, resulting in the shrinkage of muscle fibers, thereby causing a decrease in muscle cross-sectional area; activation of proteolytic systems is believed to be the primary driver of this process [2,3].

Adverse outcomes associated with sarcopenia include an increased risk of falls, frailty, and mortality [4,5]. Muscle atrophy and sarcopenia affect not only older adults but also people with chronic diseases, such as cancer, diabetes, and chronic obstructive pulmonary disease [6]. Moreover, sarcopenia is a major public health concern as the global population ages. The World Health Organization estimates that by 2050, the number of people aged 60 years or older will reach 2 billion, and the number of people aged 80 years or older will reach 426 million. The economic burden of sarcopenia is expected to increase as the number of older adults increases [4,5]. This highlights the importance of understanding the mechanisms behind muscle atrophy and sarcopenia to facilitate the development of effective interventions to prevent or mitigate these conditions.

Preventing muscle atrophy or sarcopenia can be achieved through a combination of proper nutrition and physical activity. For example, a diverse diet as well as appropriate protein intake can help to improve muscle mass and strength [7,8,9]. In addition, engaging in healthy exercises such as aerobic and resistance exercise benefits by maintaining physical functions in older adults [10,11]. Furthermore, it has been suggested that the combination of a balanced diet, healthy oral functions, adequate physical activity, and robust social engagement may be even more beneficial for preventing sarcopenia [12] as well as frailty [13]. Furthermore, in clinical therapy, non-steroidal anti-inflammatory drugs have been used to treat muscle atrophy; however, they could be associated with adverse effects and have harmful drug–drug interactions [14,15].

Spices and herbs have been found to have numerous health benefits including anti-inflammatory and anticancer effects and have shown to improve cognition as well [16,17]. In a recent study, laurel was found to have a positive impact on preventing skeletal muscle atrophy induced by dexamethasone, both in vitro and in a rat model [18]. Moreover, morroniside, extracted from the medicinal herb *Coronus officinalis*, has been considered an efficient and safe treatment for inflammatory muscle atrophy because of its potential to inhibit both canonical and non-canonical NF-κB signaling pathways, regulate inflammatory factors, and maintain the balance between anabolic and catabolic protein processes [19]. Mountain Ginseng, a traditional herbal medicine, has the capability to effectively suppress muscle atrophy by causing a significant improvement in the levels of myosin heavy chain protein in myoblasts, thereby promoting differentiation and inducing an increase in the diameter of muscle tubes and formation of muscle fibers [20]. Thus, spices and herbs show great potential in improving muscle atrophy especially because of the high safety they offer.

Moreover, as for the effects of herbal bioactive compounds on muscle atrophy, curcumin, resveratrol, epigallocatechin gallate, and astragaloside IV have been indicated to be effective against muscle atrophy. For example, curcumin is a bioactive compound found in turmeric that possesses anti-inflammatory and antioxidant properties. Several studies have investigated its effects on muscle atrophy and found that it can help prevent muscle loss by reducing inflammation and oxidative stress [21]. Another compound, resveratrol, is a polyphenol found in grapes, red wine, and other plants that has also exhibited anti-inflammatory and antioxidant effects as well as muscle loss prevention by reducing inflammation and oxidative stress [22]. Moreover, epigallocatechin gallate, a catechin found in green tea, has been found to modulate muscle homeostasis in type 2 diabetes by improving muscle lipid oxidation and stimulate glucose uptake in insulin-resistant skeletal muscle [23]. Further, astragaloside IV, a saponin found in Astragalus membranaceus, has demonstrated anti-inflammatory and antioxidant effects as well as efficient prevention of muscle loss by regulating mitochondrial quality control in a murine model of hind limb suspension [24].

In this study, we focused on turmeric, a spice commonly used in cooking and traditional medicine that has gained considerable attention in recent years owing to its potential therapeutic benefits, particularly in muscle health. Several studies have explored the effects of turmeric on muscle tissue, and the results have been promising. For example, turmeric has been found to be effective in blocking lipopolysaccharide-induced muscle loss by inhibiting p38-mediated upregulation of atrogin-1/MAFbx [25]. Additionally, a water extract of *Curcuma longa L*. has been shown to prevent muscle atrophy by modulating related genes and increasing antioxidant potential [26]. Furthermore, turmeric has shown to improve skeletal muscle atrophy in a mouse model of streptozotocin-induced diabetes, inhibiting protein ubiquitination without affecting protein synthesis [27]. In a recent study, we found that turmeric extract (TE) improved muscle atrophy by enhancing the expression of MAFbx (also known as FBXO32/Atrogin-1) and MuRF1 genes and proteins in dexamethasone-treated mice and myotubes [28].

Despite these findings, the effects of TE on age-related muscle atrophy and sarcopenia are not yet fully understood. As the global population continues to age, sarcopenia and muscle loss become increasingly relevant health concerns. Considering this, the present study aims to address the gap in knowledge by using a senescence-accelerated mouse model to investigate the effects of TE on age-related muscle atrophy. Through our results, we hope to provide valuable insights into the potential role of turmeric in maintaining muscle health and preventing age-related muscle loss.

## 2. Materials and Methods

### 2.1. Animals and Experimental Design

Twenty-six-week-old male, senescence-accelerated mouse resistant (SAMR) mice and senescence-accelerated mouse prone 8 (SAMP8) mice were housed in an individual animal facility at a temperature of 22 ± 1 °C, humidity of 60 ± 5%, and reversed light/dark cycle (12:12 h). The mice were provided with ad libitum access to both water and normal feed composed of powdered AIN-93G diet (American Institute of Nutrition, 1993). The experimental diet was supplemented with 2% TE powder [19], and corn starch was added to maintain the caloric balance of the composition of each feed (Table 1). Three groups of seven mice each were created and named as follows: (1) CON group: SAMR mice + normal diet; (2) P8 group: SAMP8 + normal diet; and (3) P8 + TE group: SAMP8 mice + 2% TE diet. All mice were anesthetized using isoflurane in the 10th week. Body weight and food intake was measured every week. Organs of the lower limb skeletal muscles (gastrocnemius, soleus, plantaris, tibialis anterior, and extensor digitorum longus), liver, kidney, and fat tissues (mesenteric fat, retroperitoneal adipose tissue, and epididymal adipose tissue) were removed, weighed, and flash-frozen for further analysis. The animal approval protocol number is P20–033. All experimental procedures and animal health care were approved by the Animal Experiment Committee of the University of Tokyo and conducted according to the University of Tokyo Experimental Procedures.

### 2.2. Total RNA Extraction for Real-Time PCR

We extracted the total RNA from the tibialis anterior, gastrocnemius, and soleus muscles using the TRIzol reagent and the RNeasy Fibrous Tissue Mini Kit (Qiagen, Hilden, Germany), which is a widely used method for isolating high-quality RNA from fibrous tissue samples. The total RNA sample (500 ng) was then reverse-transcribed into complementary DNA using a PrimeScript™ RT Master Mix (Perfect Real Time; Takara Bio Inc., Shiga, Japan) to perform real-time PCR analysis. The instructions provided by the manufacturer were followed during this process, and the PCR analysis was conducted using the Thermal Cycler Dice Real-Time System TP800 (Takara Bio Inc.) under specific conditions. The initial step was denaturation at 95 °C for 30 s and then 40 cycles of denaturation at 95 °C for 5 s, followed by annealing and extension at 60 °C for 30 s. This is the standard protocol for real-time PCR. The samples were matched to a standard curve generated by a five-fold dilution of a template that was amplified using the same real-time PCR conditions. This standard curve was used to determine the relative quantity of the target gene present in the samples. Real-time PCR data were presented as the fold-change, which is a measure of how much the target gene is expressed relative to a reference gene. In this case, the data were normalized to the mRNA expression of glyceraldehyde-3-phosphate dehydrogenase, which is a commonly used reference gene in real-time PCR experiments. The primer sequences used in this study have been mentioned in Supplementary Table S1.

### 2.3. Western Blot Analysis and Substrate-Level Phosphorylation

To assess the protein expression and phosphorylation levels in skeletal muscle tissue, we performed protein extraction, electrophoresis, and western blotting. Skeletal muscle tissue lysates were obtained and analyzed to investigate protein expression and phosphorylation levels. A total of 15–40 µg of lysates was used for this purpose. The methods for electrophoresis and blotting followed in this study were the same as those for our previous study [18]. Protein content of the samples was quantified using the Lowry assay, with bovine serum albumin (BSA) serving as the standard. Subsequently, equivalent amounts of protein samples (20 µg) were separated via sodium dodecyl sulfate-polyacrylamide gel electrophoresis and transferred onto polyvinylidene fluoride membranes. The membranes were then blocked with 5% BSA in tris-buffered saline with tween-20 (TBST) for 2 h. The primary antibodies used in this study were directed against atrogin-1/MAFbx (Abcam, Cambridge, UK; Cat. #ab168372), phospho-Akt (Ser473; Cell Signaling, Danvers, MA, USA; Cat. #9271), and Akt (Cell Signaling; Cat. #9272). These primary antibodies were incubated with the membranes overnight at 4 °C. Following this, the membranes were washed thrice with TBST and incubated with the recommended dilution of the conjugated secondary antibodies in TBST for 2 h at room temperature. The binding of the antibodies to the target proteins was visualized using an ECL Western Blotting Detection System (GE HealthCare, Chicago, IL, USA), and ECL signals were quantified using Ez-Capture MG (ATTO, Tokyo, Japan). The protein expression was quantitatively analyzed using the CS Analyzer software (version 3.0, ATTO).

The following primary antibodies (all diluted 1:1000) were used: anti-MAFbx (#ab168372; Abcam), anti-MuRF1 (#sc-398608; Santa Cruz Biotechnology, Dallas, TX, USA), anti-Rheb (#13879; Cell Signaling Technology), anti-Tubulin (#3873; Cell Signaling Technology), anti-mTOR (#2983; Cell Signaling Technology), anti-Akt (#9272; Cell Signaling Technology), anti-Phospho-mTOR (Ser2448) (#5536; Cell Signaling Technology), anti-Phospho-Akt (Ser473) (#9271; Cell Signaling Technology), anti-Phospho-p70S6 Kinase (Thr389) (#9234; Cell Signaling Technology), anti-Phospho-FoxO1 (Ser256) (#9461; Cell Signaling Technology), anti-Phospho-IRS-1 (Ser612) (#2386; Cell Signaling Technology), anti-Phospho-IRS-1 (Ser307) (#07-247; MilliporeSigma, Burlington, MA, USA), anti-IRS-1 (#sc-559; Santa Cruz Biotechnology), anti-Phospho-NF-κB p65 antibody (Ser536) (#3033; Cell Signaling Technology), anti-Phospho-IκBα (Ser32) (#2859; Cell Signaling Technology), and anti-IκBα (#4814; Cell Signaling Technology). The secondary antibodies anti-rabbit IgG (#NA934) and anti-mouse IgG (#NA931) were purchased from GE HealthCare.

### 2.4. Statistical Analysis

The mean and standard error (SE) were calculated for the data. One-way analysis of variance was used in the statistical analysis, followed by Tukey’s test. The cutoff for statistical significance was *p* < 0.05. The statistical difference between three groups was expressed using the letters ‘a’ and ‘b’. If the same letter was used between two groups, it meant that there was no statistical difference.

## 3. Results

### 3.1. Food Intake and Body Weight

**Figure 1:**
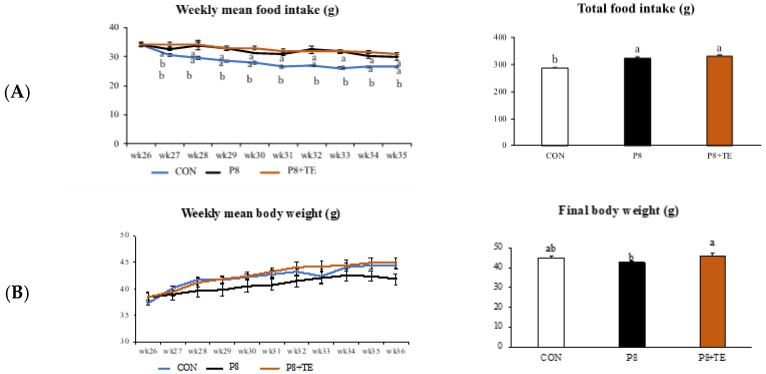
General characteristics of CON, P8, and P8-TE groups from 26th to 36th week. (**A**) Weekly mean food intake and total food intake for all groups. (**B**) Weekly mean body weight and final body weight for all groups. All values are mean ± standard error (SE) (n = 6–7) via Tukey’s test. (CON, control: SAMR mice with normal diet; P8, SAMP8 mice with normal diet; P8 + TE, SAMP8 mice with turmeric extract (TE)-supplemented diet).

With respect to weekly and total mean food intake from week 26 to 36, no significant differences were observed between the P8 + TE and P8 groups, but both groups showed different results compared to the CON group (Figure 1A). No significant difference was observed in weekly mean body weight between the three groups; however, the final body weight (week 36) was different between the P8 + TE and P8 groups, although they showed no significant differences compared to the CON group (Figure 1B).

### 3.2. Fat Mass, Organ Weight, and Muscle Mass

**Figure 2:**
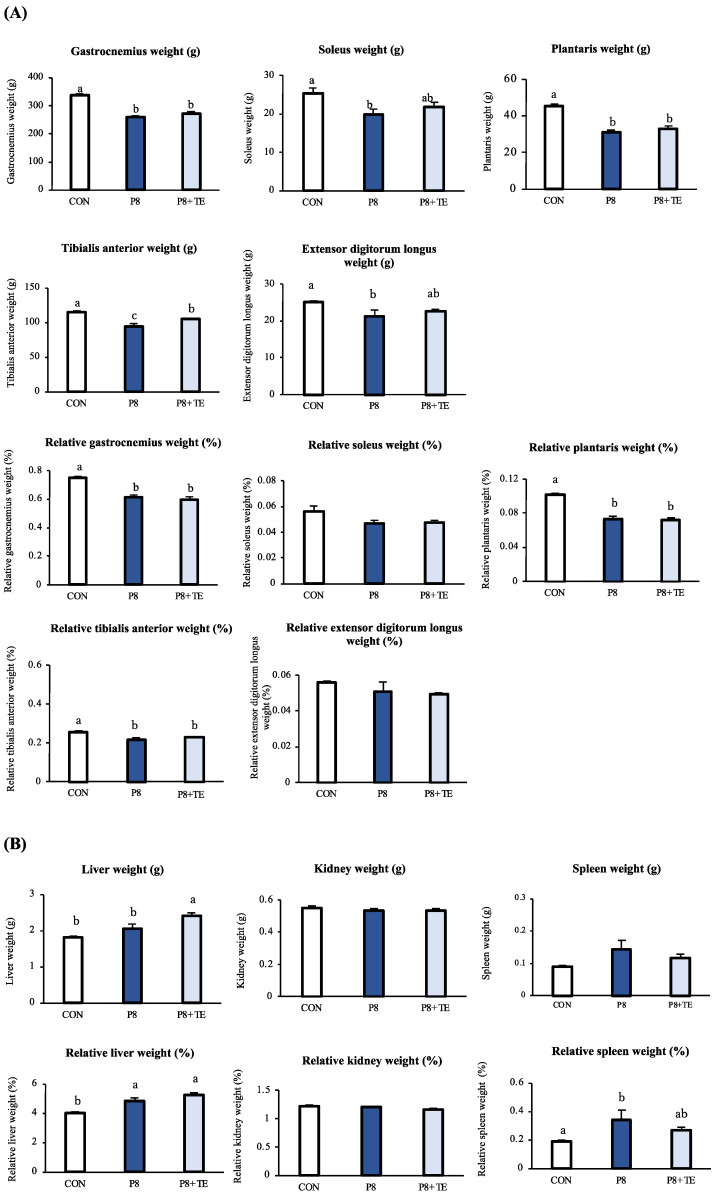

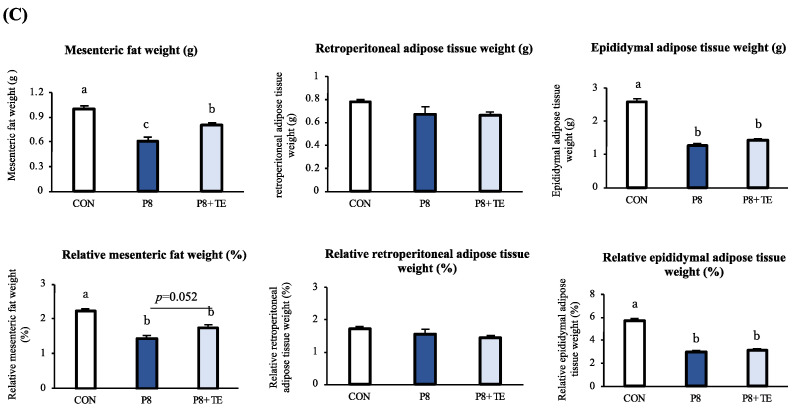
Changes in fat mass, organ weight, and muscle mass of the mice across CON, P8, and P8-TE groups. (**A**) Weight (g) and relative weight (by weight, %) of gastrocnemius, soleus, plantaris, tibialis anterior, and extensor digitorum longus. (**B**) Weight (g) and relative weight (by weight, %) of liver, kidney, and spleen. (**C**) Weight (g) and relative weight (by weight, %) of mesenteric fat, retroperitoneal adipose tissue, and epididymal adipose tissue. All values are mean ± SE (n = 6–7) via Tukey’s test.

After 36 weeks of feed treatment, the tibialis anterior muscle weight was significantly different between the P8 + TE and P8 groups, as well as between the P8 + TE and CON groups. However, no significant differences were observed among the other (relative) muscle weights (Figure 2A). Regarding organ weight, the liver weight in the P8 group was significantly different from the CON and P8 + TE groups. Moreover, the relative liver weight in the CON group was significantly different from the P8 and P8 + TE groups. However, no significant differences were observed in the (relative) kidney/spleen weights (Figure 2B). Regarding fat tissues, the P8 and P8 + TE groups showed slight differences (*p* = 0.052) in (relative) mesenteric fat tissue weight. No difference was found in retroperitoneal adipose or epididymal adipose tissue (Figure 2C).

### 3.3. mRNA Expression in Skeletal Muscles

**Figure 3:**
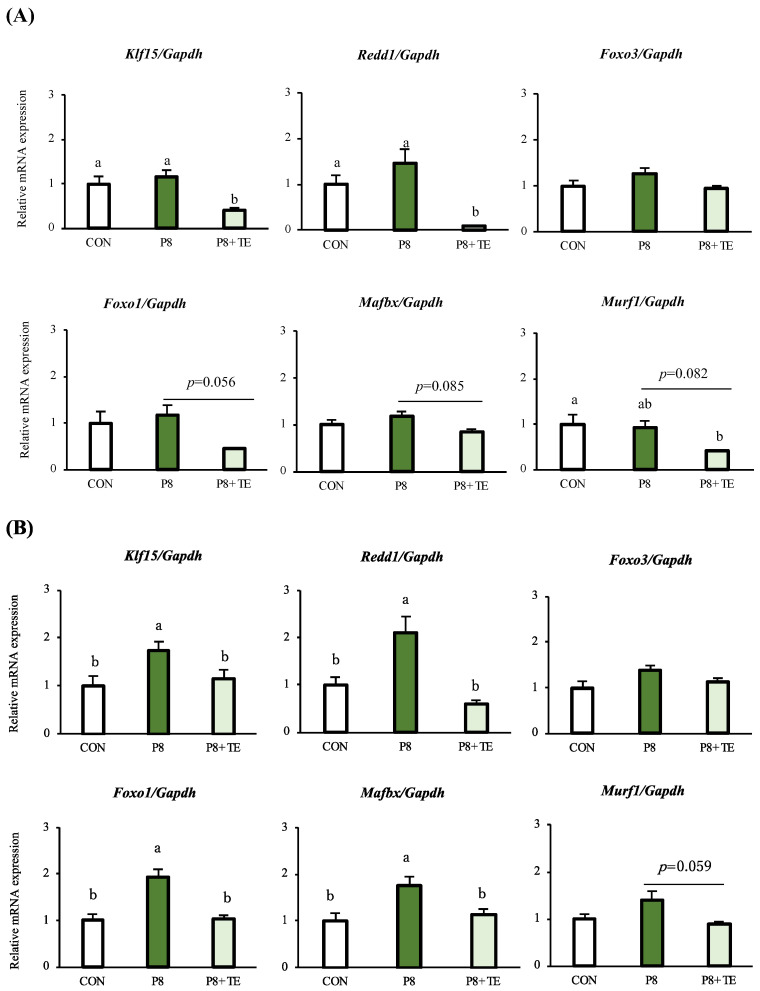

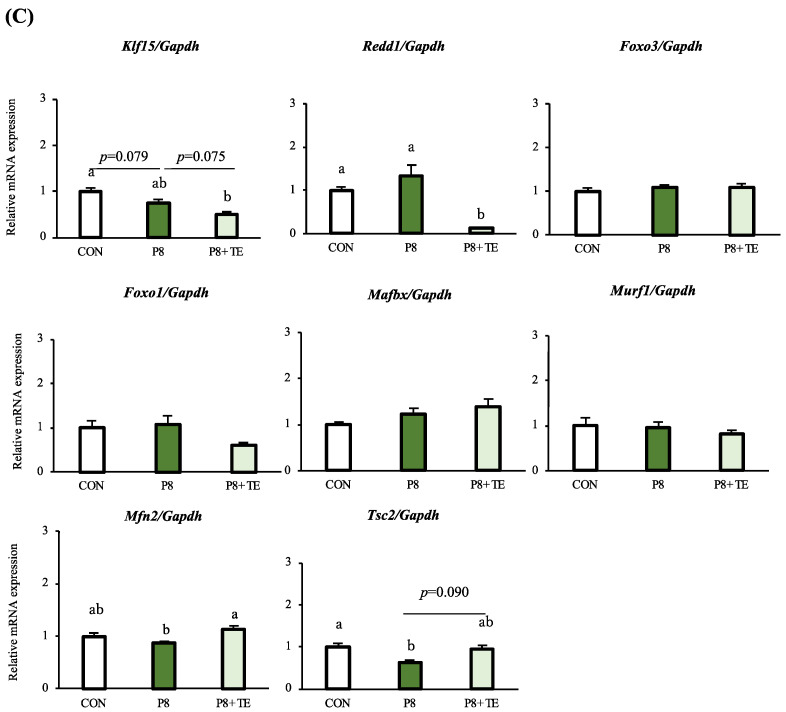
Changes in mRNA expression in skeletal muscles of the CON, P8, and P8-TE groups. (**A**) Gastrocnemius mRNA expression of genes. (**B**) Soleus mRNA expression of genes. (**C**) Tibialis anterior mRNA expression of genes. All values are mean ± SE (n = 6–7) via Tukey’s test.

TE extract significantly improved muscle atrophy-related mRNA expression, especially in the glucocorticoid receptor-FoxO signaling pathway in skeletal muscle, including muscle protein synthesis-relate gene of DNA-damage-inducible transcript 4 (*redd1*), mRNA expression levels of muscle atrophy-related gene Ruppel-like factor 15 (*klf15*), FoxO3-induced autophagy-related gene of forkhead box protein O1 (*foxo1*), myosin ubiquitination and degradation-related gene of tripartite motif-containing 63 (*murf1*), and muscle atrophy-related f-box protein 32 (*mafbx*), in the gastrocnemius, soleus, and tibialis anterior tissues. In both the gastrocnemius and soleus tissues, *klf15*, *redd1*, *foxo1*, *mafbx*, and *murf1* were downregulated in the P8 + TE group compared to the P8 group (Figure 3A,B). In the tibialis anterior tissue, the gene *klf15* showed a downregulation trend, and *redd1* was significantly downregulated in the P8 + TE compared to the P8 group. Furthermore, the cell growth and proliferation-related gene of tuberous sclerosis complex subunit 2 (*tsc2*) showed an upregulation trend, and the muscle mitochondrial damage-related mitofusin 2 (*mfn2*) was significantly upregulated in the P8 + TE group compared to the P8 group (Figure 3C).

### 3.4. Protein Expression and Substrate-Level Phosphorylation in Skeletal Muscles

We measured the expression of skeletal muscle proteins MAFbx, MuRF1, and Rheb in the tibialis anterior and MAFbx and MuRF1 in the soleus; however, there were no significant differences in their expression levels between the P8 and P8 + TE groups (Figure S1). Moreover, we detected substrate-level phosphorylation in tibialis anterior proteins, including mammalian target of rapamycin complex 1 (mTORC1), protein kinase B (Akt), 70 kDa ribosomal protein S6 kinase 1 (p70S6K1), FoxO1, insulin receptor substrate (IRS) proteins at serine^612^ and serine^307^ (IRS-1Ser^612^ and IRS-1Ser^307^), nuclear factor-kappa B (NF-κB), and nuclear factor of kappa light polypeptide gene enhancer in B-cells inhibitor alpha (IκBα). However, TE supplementation did not cause significant differences in the levels of p-Akt, p70S6K1, and 4E-BP1 between the P8 and P8 + TE groups (Figure S2).

## 4. Discussion

Muscle atrophy, accompanied with loss of muscle mass and strength, is a widespread condition that affects individuals as they age. Muscle function decline is not only an aesthetic concern but also has serious implications for health and well-being. Muscle atrophy is associated with a range of age-related illnesses, including but not limited to sarcopenia, which is the age-related decline in muscle mass, strength, and function, as well as frailty, which is a state of increased vulnerability to falls and other adverse health outcomes owing to decreased physical and cognitive reserve [29,30]. However, its mechanism, especially with respect to age-related aspects, remains unclear. In our previous study, we explored the impact of TE on muscle atrophy in dexamethasone-treated mice and found that it produced a significant effect by suppressing the expression of MAFbx and MuRF1 in C2C12 cells and skeletal muscles of mice [28]. Encouraged by these results, we expanded our investigation to examine the effect of TE on age-related muscle atrophy.

According to previous research, SAMP8 mice display a faster rate of muscle aging than SAMR mice, which is reflected through their 40% shorter median lifespan and double the grading score of senescence at the age of 8 months [31,32]. Furthermore, it has been reported that age-related muscle mass loss, selective type-II fiber atrophy, and slower contraction speeds occur at a relatively younger age in the soleus muscle of SAMP8 mice compared with other mouse strains; furthermore, the muscle atrophy is also more pronounced in SAMP8 than in SAMP6 and SAMR1 aged mice [33].

Moreover, previous research has described the molecular biological modifications that occur with aging in the fast-twitch extensor digitorum longus muscle of the SAMP8 mouse model using capillary electrophoresis-mass spectrometry [34]. Furthermore, the examination of the gastrocnemius muscle in SAMP8 mice revealed a peak in muscle mass at 7 months, followed by a decline in both functional and structural aspects at 8 months [35]. Hence, we used the two senescence-accelerated mouse models to investigate the effect of TE on age-related muscle atrophy in the present study. We found that TE supplementation significantly improved body weight loss at 36 weeks when comparing the P8 + TE and P8 groups. Notably, there was no statistical difference in the total food intake between P8 + TE and P8 groups, which suggested that the observed effect of TE on body weight loss was not because of differences in food intake, a finding that further validates our results (Figure 1). Moreover, tibialis anterior weight, mesenteric fat tissue weight, and relative mesenteric fat tissue also significantly or marginally improved in the P8 + TE group compared to the P8 group.

**Figure 4:**
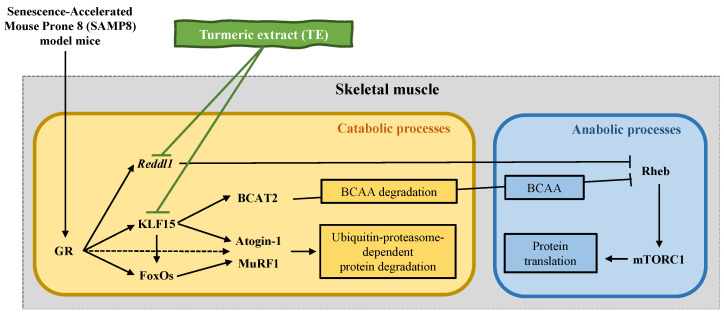
Schematic representation of the mechanism underlying muscle atrophy attenuation in the Senescence-Accelerated Mouse Prone 8 (SAMP8) model mice via TE. TE has been shown to protect against SAMP-induced skeletal muscle atrophy by inhibiting the upregulation of MuRF1 mRNA and regulating the mRNA expression of *redd1*, *klf15*, *foxo1*, *murf1*, and *mafbx* in the muscles of mice. Additionally, it is believed that the inhibition of the binding of glucocorticoid receptor (GR) or FoxO1 to the glucocorticoid response element (GRE) or FOXO-binding element (FBE) in the MuRF1 promoter in the skeletal muscle might contribute to the beneficial effects of TE.

In terms of transcriptomics, TE supplementation improved muscle atrophy in fast-twitch muscle fibers (gastrocnemius and tibialis anterior), which contract powerfully over short durations and fatigue quickly, as well as in slow-twitch muscle fibers (soleus), which are fatigue-resistant and produce sustained, small movements and postural control [36]. The mRNA expression of *klf15* in the gastrocnemius and *redd1* in the soleus were significantly upregulated in P8 compared to the CON group; moreover, both genes were significantly downregulated in the muscle tissue of the P8 + TE group compared to the P8 group, while only *redd1* was significantly downregulated in the tibialis anterior. The mRNA expression of *foxo1* in the soleus was also significantly downregulated in the P8 + TE group compared to the P8 group. These data corroborate our previous study that investigated the effects of TE supplementation on improving muscle atrophy in dexamethasone-treated mice [28]. Further, *klf15* is a member of the zinc finger transcription factor family that can induce the transcription of muscle atrophy-related genes, such as *marfbx1* and *murf1* through *foxo3a* [37]. In this study, only the *marfbx1* mRNA expression was significantly downregulated in the soleus of the P8 + TE group compared to the P8 group. According to a previous study, the ubiquitin ligase MuRF1 is associated with the ubiquitination and degradation of myosin, which occurs by binding with the glucocorticoid receptor (GR) and transcription factors FoxO and Klf15 [20]. FoxO3 is associated with the expression of atrophy and autophagy-related genes as well as autophagic-lysosomal and ubiquitin-proteasomal pathways that control protein degradation [38]. Moreover, a related study has reported that FoxO deletion improves muscular strength and preserves muscular strength in part by mildly suppressing atrophic pathways, including the regulation of Gadd45a and Ube4a expression, without causing autophagosome accumulation in muscles in old age [39]. In this study, although *foxo3a* expression showed no significant change among the three groups, *foxo1*, which promotes FoxO3-induced autophagy [40], was marginally or significantly downregulated in the P8 + TE group compared to the P8 group in the gastrocnemius (*p* = 0.056) and soleus (*p* < 0.05), respectively (Figure 3). TE supplementation also increased the soleus expression of *redd1*, which is a repressor of mTORC expression and related to the nutrient-induced response of muscle protein synthesis. Its expression is upregulated under pathological conditions related to muscle atrophy and downregulated under hypertrophic conditions [41,42]. Furthermore, the *redd1* gene is now known to be a negative regulator of muscle mass through inhibition of the Akt/mTORC1 signaling pathway, which controls both skeletal muscle mass and metabolism by stimulating various ATP-demanding activities, such as protein synthesis, glycogen storage, and mitochondrial biogenesis [43]. This can somewhat explain how TE supplementation improved muscle mass and muscle strength in the present study. These datasets may reveal the main mechanism of TE supplementation in age-related muscle atrophy. TE supplementation was previously shown to protect against age-related skeletal muscle atrophy by improving the mRNA expression of *redd1*, *klf15*, *foxo1*, *murf1*, and *mafbx* in SAMP8 mice in the GR-FoxO signaling pathway [44,45], which is involved in a variety of downstream molecular cascades toward muscle atrophy and is related to mTOR activity [37,46]. Moreover, TE supplementation inhibited the binding of two transcription factors, GR and FoxO1, to specific regulatory elements in the MuRF1 promoter in skeletal muscle, thereby maintaining the dynamic balance between anabolic and catabolic processes in fast/slow-twitch muscles (Figure 4).

In the tibialis anterior tissue, *mfn2* mRNA expression was significantly upregulated in the P8 + TE group compared to the P8 group. The gene *mfn2* controls muscle mitochondrial damage, and its age-related decrease in muscles is a determinant of the inhibition of mitophagy and accumulation of damaged mitochondria, triggering the induction of muscle atrophy and even sarcopenia [47]. In the present study, the significant upregulation of *mfn2* meant that TE supplementation may have improved age-related mitochondrial damage or fusion in muscle tissues and improved muscle atrophy. Another gene, *tsc2*, which produces tuberin protein to help control cell growth, division (proliferation), and size in muscle cells, was also slightly upregulated (*p* = 0.09) in the tibialis anterior because of TE supplementation [48]. According to a previous study, decreased *tsc2* expression in mouse skeletal muscle caused reduced muscle mass, possibly because *tsc2* is associated with hTSC1 proteins, which maintain a relatively normal response to insulin signaling in muscle tissues [48].

Proteomics provide valuable insights into the molecular mechanisms underlying age-related muscle atrophy. One such area of interest is the degradation of muscle tissue, in which two E3 ubiquitin ligase proteins, MAFbx and MuRF1, have been identified as key players [49]. The protein MAFbx, also known as Atrogin-1, is involved in the degradation of major muscle proteins and has been shown to target the transcription factor MyoD and protein synthesis activator eIF3f, both of which play critical roles in muscle synthesis. Thus, the degradation of these proteins by MAFbx contributes to the overall loss of muscle mass [50]. In contrast, the protein MuRF1 was originally identified as a muscle-specific RING finger protein that binds to the kinase domain of titin, a giant sarcomeric protein related to the regulation of muscle mass. Further studies have revealed that MuRF1 is involved in the degradation of several key muscle proteins, and its activity has been linked to various muscle wasting diseases, such as muscular dystrophy [51]. Additionally, the protein Rheb was found to be related to the activation of the mTOR signaling pathway, which is a key regulator of muscle mass [52]. Our proteomic analysis showed that there was no significant difference in the protein expression of MAFbx, MuRF1, and Rheb in the tibialis anterior muscle and no significant difference in the protein expression of MAFbx and MuRF1 in the soleus muscle (Figure S1). These results were unexpected and diverged from our previous findings based on gene expression analysis. This may be because there is a time lag for mRNA expression to affect proteins such that mRNA levels may not fully (40–70%) reflect the abundance of the corresponding proteins. This time lag (delay) between the synthesis of mRNA from DNA and the translation of mRNA into a protein is caused by several factors, including the time required for mRNA to be transcribed and processed in the nucleus, exported from the nucleus to the cytoplasm, and translated into protein [53].

Furthermore, regarding mTORC1-mediated protein synthesis, the phosphorylation levels of the key substrates of the kinase p70S6K1 (S6K1) and eIF4E-binding protein 1 (4EBP1) are associated with the activation status of mTORC1 [54,55]. Therefore, to test mTORC1 activity and metabolic reaction in skeletal muscle proteins leading to the production of adenosine triphosphate or guanosine triphosphate [56], we further detected the substrate-level phosphorylation in tibialis anterior proteins, such as the regulation of protein synthesis and degradation-related protein Akt [52] and mTORC1 [57], a kinase enzyme protein regulating muscle mass, p70S6K1 [58], and regulator protein related to skeletal muscle atrophy of Foxo1 [59] and the suppression of Akt activity-related protein IRS-1Ser^612^ [60], c-Jun NH2-terminal kinase activity in atrophic skeletal muscle-related protein IRS-1Ser^307^ [61], an important molecular target protein for the prevention of skeletal muscle, NF-κB [62], and the protein related to NF-κB activity and fiber atrophy in muscle, IκBα [63]. However, TE supplementation did not cause significant effects among the three groups (Figure S2).

According to previous research, there are three major ingredients in turmeric, which are curcuminoids: curcumin (a primary constituent responsible for yellow color of turmeric), desmethoxycurcumin, and bisdemethoxycurcumin [40]. Among these, curcumin was indicated to be obviously effective in ameliorating muscle atrophy. For example, curcumin alleviates lipopolysaccharide-induced muscle loss by inhibiting the p38-mediated upregulation of atrogin-1/MAFbx [16]. Moreover, recent studies have revealed that curcumin, a polyphenol found in the spice turmeric, holds potential as an effective therapy for attenuating skeletal muscle atrophy in diabetic mice owing to its ability to relieve inflammation, protein ubiquitination, and oxidative stress [18]. Curcumin improved sepsis-induced muscle atrophy by blocking the increase in muscle protein breakdown; furthermore, it reduced the nuclear translocation and DNA-binding activity of the NF-kB subunit p65 [41]. Therefore, in this study, we speculated that curcumin played a major role as the active ingredient in turmeric by preventing muscle atrophy in senescence-accelerated mice. However, this study had certain limitations. First, we did not investigate the effects of precise and powerful constituent of TE on aging-related muscular atrophy. Second, although significant differences were observed on transcriptomic data, the analysis from other viewpoints of analysis such as metabolomics and metagenomics are indeed necessary to further investigate the impact of TE on the age-related muscle atrophy. Third, the clinical study is quite necessary to further investigate the effects of TE on muscle atrophy in older adults.

## 5. Conclusions

The present study aimed to examine the effect of TE supplementation on age-related muscle atrophy in a senescence-accelerated mouse model. TE supplementation had certain effects on ameliorating the decrease in body weight, tibialis anterior weight, and (relative) mesenteric fat tissue weight in SAMP8 mice. Additionally, TE supplementation might suppress the binding of GR or FoxO1 to the glucocorticoid response element or FoxO-binding element in the promoter region of the muscle-specific ubiquitin ligase gene, MuRF1, in skeletal muscle. This inhibition might lead to a favorable shift in the balance between anabolic and catabolic processes, thereby promoting muscle mass and strength. This may also offer the insights into TE’s potential as a therapeutic intervention for sarcopenia and muscle atrophy. Especially in the super-aging society of Japan, sarcopenia is a significant health concern related to various outcomes. The present study indicated the potential of TE on preventing age-related muscle atrophy and sarcopenia. However, further research is needed to confirm these findings in humans and determine the optimal dose and duration of TE supplementation. In addition to its potential application in preventing age-related muscle atrophy, TE has also been studied for its potential benefits in other medical uses. For example, TE has been shown to have anti-inflammatory and anti-cancer properties as well as potential benefits for cardiovascular health and male reproductive function. Overall, the findings of this study suggest that TE might be able to contribute to the prevention for age-related muscle atrophy and sarcopenia.

## Figures and Tables

**Table 1:** Composition of control diet (AIN93G) and 2% turmeric extract (TE) powder-supplemented diet.

Component	Control Diet (%)	2% TE-Supplemented Diet (%)
Casein	20.00	20.00
ꞵ-corn starch	39.75	37.75
α-corn starch	13.20	13.20
Soybean oil	7.00	7.00
Sucrose	10.25	10.25
Cellulose	5.00	5.00
Vitamin mixture	1.00	1.00
Mineral mixture	3.50	3.50
L-cystine	0.30	0.30
TE powder	0.00	2.00

## Data Availability

Not applicable.

## Supplementary Materials

The following supporting information can be downloaded from https://www.mdpi.com/article/10.3390/life13040941/s1, Table S1: Oligonucleotide sequences used in the real-time PCR analysis; Figure S1: (A) Tibialis anterior protein expression of MAFbx, MuRF1, and Rheb, analyzed using western blotting; (B) Soleus protein expression of MAFbx and MuRF1 analyzed via western blotting. All values are mean ± SE (n = 6–7) via Tukey’s test; Figure S2: Substrate-level phosphorylation of the tibialis anterior proteins. All values are mean ± SE (n = 6–7) via Tukey’s test.
