# Supplementary material for: Effects of Turmeric Extract on Age-Related Skeletal Muscle Atrophy in Senescence-Accelerated Mice
Source: Life (Basel). 2023 Apr 3;13(4):941. doi: 10.3390/life13040941 (PMC10141758; doi:10.3390/life13040941)
Supplement: Supplementary file 1 [file life-13-00941-s001.zip › life-2209352-supplementary.docx]

**Table S1.** Oligonucleotide sequences used in the real-time PCR analysis.

| Gene |  | Sequence |
| --- | --- | --- |
| *gapdh* | Forward | 5'-TGTGTCCGTCGTGGATCTGA-3' |
|  | Reverse | 5'-TTGCTGTTGAAGTCGCAGGAG-3' |
|  |  |  |
| *mafbx* | Forward | 5'-GCTGGTGGGCAACATTAACA-3' |
|  | Reverse | 5'-GTTGTAAGCACACAGGCAGGTC-3' |
|  |  |  |
| *murf1* | Forward | 5'-CTGAGTAACTGCATCTCCATGCT-3' |
|  | Reverse | 5'-TCCTTCACCTGGTGGCTATTC-3' |
|  |  |  |
| *klf15* | Forward | 5'-CCAAGAGCAGCCACCTCCAA-3' |
|  | Reverse | 5'-CAACTCATCTGAGCGGGAAA-3' |
|  |  |  |
| *foxo1* | Forward | 5'-ACATTTCGTCCTCGAACCAGCTCA-3' |
|  | Reverse | 5'-ATTTCAGACAGACTGGGCAGCGTA-3' |
|  |  |  |
| *foxo3* | Forward | 5'-TCGTCTCTGAACTCCTTGCGT-3' |
|  | Reverse | 5'-TGGAGTGTCTGGTTGCCGT-3' |
|  |  |  |
| *redd1* | Forward | 5'-CCAGAGAAGAGGGCCTTGA-3' |
|  | Reverse | 5'-CCATCCAGGTATGAGGAGTCTT-3' |
|  |  |  |
| *tsc2* | Forward | 5'-CCACAGCGCCATCTACAACA-3' |
|  | Reverse | 5'-TGCCATCCCTACAAAGAACACA-3' |
|  |  |  |
| *lc3* | Forward | 5'-CGATACAAGGGGGAGAAGCA-3' |
|  | Reverse | 5'-ACTTCGGAGATGGGAGTGGA-3' |

**(A)**

MAFbx/α-Tubulin

|  | MAFbx/α-Tubulin (A.U.) |
| --- | --- |
| CON | 1 |
| P8 | 1.02065 |
| P8+TE | 1.03837 |

MAFbx

CON

P8

P8+TE

α-Tubulin

CON

P8

P8+TE

CON

P8

P8+TE

CON

P8

P8+TE

CON

P8

P8+TE

MuRF1

α-Tubulin

CON

P8

P8+TE

CON

P8

P8+TE

CON

P8

P8+TE

MuRF1/α-Tubulin

|  | MuRF1/α-Tubulin (A.U.) |
| --- | --- |
| CON | 1 |
| P8 | 0.985334 |
| P8+TE | 0.929972 |

α-Tubulin

Rheb

CON

P8

P8+TE

CON

P8

P8+TE

CON

P8

P8+TE

CON

P8

P8+TE

Rheb/α-Tubulin

|  | Rheb/α-Tubulin (A.U.) |
| --- | --- |
| CON | 1 |
| P8 | 1.07739 |
| P8+TE | 1.19371 |

**(B)**

CON

P8

P8+TE

MAFbx

CON

P8

P8+TE

CON

P8

P8+TE

α-Tubulin

CON

P8

P8+TE

MAFbx/α-Tubulin

|  | MAFbx/α-Tubulin (A.U.) |
| --- | --- |
| CON | 1 |
| P8 | 1.28733 |
| P8+TE | 1.26478 |

MuRF1/α-Tubulin

|  | MuRF1/α-Tubulin (A.U.) |
| --- | --- |
| CON | 1 |
| P8 | 0.854005 |
| P8+TE | 0.940681 |

CON

P8

P8+TE

MuRF1

α-Tubulin

CON

P8

P8+TE

CON

P8

P8+TE

CON

P8

P8+TE

**Figure S1.** (**A**) Tibialis anterior protein expression of MAFbx, MuRF1, and Rheb analyzed using western blotting; (**B**) Soleus protein expression of MAFbx and MuRF1 analyzed using western blotting. All values are mean ± standard error (n = 6–7) via Tukey’s test.

p-Akt

Akt

CON

P8

P8+TE

CON

P8

P8+TE

CON

P8

P8+TE

CON

P8

P8+TE

mTOR

p-mTOR

CON

P8

P8+TE

CON

P8

P8+TE

CON

P8

P8+TE

CON

P8

P8+TE

p-Akt/Akt

|  | p-Akt/Akt (A.U.) |
| --- | --- |
| CON | 1 |
| P8 | 0.553776 |
| P8+TE | 1.21943 |

p-mTOR/mTOR

|  | p-mTOR/mTOR (A.U.) |
| --- | --- |
| CON | 1 |
| P8 | 1.05771 |
| P8+TE | 0.898957 |

p-p70S6K

p70S6K

CON

P8

P8+TE

CON

P8

P8+TE

CON

P8

P8+TE

CON

P8

P8+TE

p-FoxO1/FoxO1

|  | p-FoxO1/FoxO1 (A.U.) |
| --- | --- |
| CON | 1 |
| P8 | 1.3838 |
| P8+TE | 1.14297 |

p-70S6K/p70S6K

|  | p-70S6K/p70S6K (A.U.) |
| --- | --- |
| CON | 1 |
| P8 | 1.07678 |
| P8+TE | 1.07133 |

p-FoxO1

FoxO1

CON

P8

P8+TE

CON

P8

P8+TE

CON

P8

P8+TE

CON

P8

P8+TE

p-IRS-1Ser307/IRS-1

|  | p-IRS-1Ser307/IRS-1 (A.U.) |
| --- | --- |
| CON | 1 |
| P8 | 0.813656 |
| P8+TE | 0.747203 |

p-IRS-1Ser612/IRS-1

|  | p-IRS-1Ser612/IRS-1 (A.U.) |
| --- | --- |
| CON | 1 |
| P8 | 1.53568 |
| P8+TE | 1.29377 |

IRS-1

p-IRS-1^Ser307^

CON

P8

P8+TE

CON

P8

P8+TE

CON

P8

P8+TE

CON

P8

P8+TE

p-IRS-1^Ser612^

IRS-1

CON

P8

P8+TE

CON

P8

P8+TE

CON

P8

P8+TE

CON

P8

P8+TE

p-IκBα/IκBα

|  | p-IκBα/IκBα (A.U.) |
| --- | --- |
| CON | 1 |
| P8 | 0.857326 |
| P8+TE | 0.820671 |

p-NF-κB/NF-κB

|  | p-NF-κB/NF-κB (A.U.) |
| --- | --- |
| CON | 1 |
| P8 | 0.648573 |
| P8+TE | 0.76656 |

CON

P8

P8+TE

p-IκBα

IκBα

CON

P8

P8+TE

CON

P8

P8+TE

CON

P8

P8+TE

p-NF-κB

NF-κB

CON

P8

P8+TE

CON

P8

P8+TE

CON

P8

P8+TE

CON

P8

P8+TE

**Figure S2.** Substrate-level phosphorylation of the tibialis anterior proteins. All values are mean ± standard error (n = 6–7) via Tukey’s test.
